# Supplementary material for: Superinjection of Holes in Homojunction Diodes Based on Wide-Bandgap Semiconductors
Source: Materials (Basel). 2019 Jun 19;12(12):1972. doi: 10.3390/ma12121972 (PMC6631951; doi:10.3390/ma12121972)
Supplement: Supplementary file 1 [file materials-12-01972-s001.pdf]

materials

MDPI

Article

Superinjection of Holes in Homojunction Diodes Based on Wide-Bandgap Semiconductors

Igor A. Khramtsov and Dmitry Yu. Fedyanin *

Laboratory of Nanooptics and Plasmonics, Moscow Institute of Physics and Technology, Dolgoprudny 141700, Russian Federation; khramtsov@phystech.edu

* Correspondence: dmitry.fedyanin@phystech.edu

Supplementary Materials

Table S1. Main material parameters used in the numerical simulations.

Diode	4H-SiC	3C-SiC	wz-ZnS	wz-AlN
Energy bandgap at T=300 K, eV	3.23 [1]	2.36 [1]	3.8 [2]	6.23 [3]
Dielectric constant	9.78 [4]	9.72 [5]	8.32 [2]	9.14 [6]
Density of states effective electron mass	$0.77m_0$ [7]	$0.72m_0$ [8]	$0.31m_0$ [2]	$0.31m_0$ [3]
Density of states effective hole mass	$0.91m_0$ [9]	$1.11m_0$ [10]	$0.7m_0$ [11]	$7.26m_0$ [12]
Acceptor concentration in the p-type region, cm^{-3}	10^{18}	10^{18}	10^{18}	10^{18}
Acceptor compensation ratio	5%	5%	5%	40% (fitted from the experimental data) [13]
Acceptor ionization energy, eV	0.23 (Al) [14]<br>0.32 (B) [15]	0.26 (Al) [16]<br>0.34 (Ga) [16]	0.6 (Cu) [17]	0.63 (Mg) [13]
Donor concentration in the n-type region, cm^{-3}	10^{18}	10^{18}	10^{18}	10^{18}
Donor compensation ratio	5%	5%	5%	10 % [18]
Donor ionization energy, eV	0.06 (N) [19]	0.05 (N) [16]	0.1 (Al) [20]	0.25 (Si) [21]

**Electron mobility in the p-type region, cm^2/Vs**	500 [22,23]	200 [24]	100 [2]	100 [21]
**Hole mobility in the p-type region, cm^2/Vs**	140 [22,23]	55 [25]	10 [26]	5 [25]
**Electron mobility in the i-type region, cm^2/Vs**	900 [22,23]	650 [24]	140 [2]	300 [25]
**Hole mobility in the i-type region, cm^2/Vs**	140 [22,23]	70 [25]	15 [26]	14 [25]
**Electron mobility in the n-type region, cm^2/Vs**	300 [22,23]	200 [24]	100 [2]	100 [21]
**Hole mobility in the n-type region, cm^2/Vs**	120 [22,23]	55 [25]	10 [26]	5 [25]
**Electron saturation velocity, cm/s**	2.2×10^7 [27]	2.0×10^7 [1]	1.8×10^7 (ZnSe value) [28]	1.5×10^7 [25]
**Hole saturation velocity, cm/s**	1.5×10^7 [1]	1.5×10^7 [1]	1.8×10^7 (the same as for electrons)	1.25×10^7 [25]
**Electron and hole recombination lifetimes in the p-type region, ns**	17[a)]	80 [29]	100 (estimated from Refs. [30–32])	10 (GaN value) [33,34]
**Electron and hole recombination lifetimes in the i-type region, ns**	20[a)]	100 [29]	100 (estimated from Refs. [30–32])	10 (GaN value) [33,34]
**Electron and hole recombination lifetimes in the n-type region, ns**	17[a)]	80 [29]	100 (estimated from Refs. [30–32])	10 (GaN value) [33,34]
**Radiative recombination coefficient, cm^3/s**	-	-	7.1×10^{-10} (ZnTe value) [35]	0.45×10^{-10} [36]

a) The recombination lifetimes for electrons and holes are calculated using the Scharfetter relation [37,38]:.

$$\tau = \frac{\tau_0}{1 + \frac{N_D + N_A}{N_{ref}}}$$

where N_D and N_A are the concentrations of donors and acceptors, respectively, N_{ref} is estimated to be 5×10^{18} cm^{-3} [38] and $\tau_0 = 20$ ns [38].

Table S2. Temperature dependence of the main material parameters of 4H-SiC.

	$T = 200$ K	$T = 400$ K
**Energy bandgap, eV**	$E_g(T) = E_g(0) - 6.5\times10^{-4}T^2/(1300+T)$ [1]	
**Electron mobility in the p-type region, cm^2/Vs**	730 [22,23]	260 [22,23]
**Hole mobility in the p-type region, cm^2/Vs**	450 [22,23]	70 [22,23]
**Electron mobility in the i-type region, cm^2/Vs**	1550 [22,23]	310 [22,23]
**Hole mobility in the i-type region, cm^2/Vs**	345 [22,23]	75 [22,23]
**Electron mobility in the n-type region, cm^2/Vs**	400 [22,23]	200 [22,23]
**Hole mobility in the n-type region, cm^2/Vs**	245 [22,23]	70 [22,23]
**SRH recombination lifetime, ns**	$\tau_{SRH} \sim (T/300)^2$ [39,40]	
**Electron saturation velocity, cm/s**	$V_{satn} \sim (T/300)^{-0.44}$ [27,41,42]	
**Hole saturation velocity, cm/s**	The same temperature dependence as for electrons	

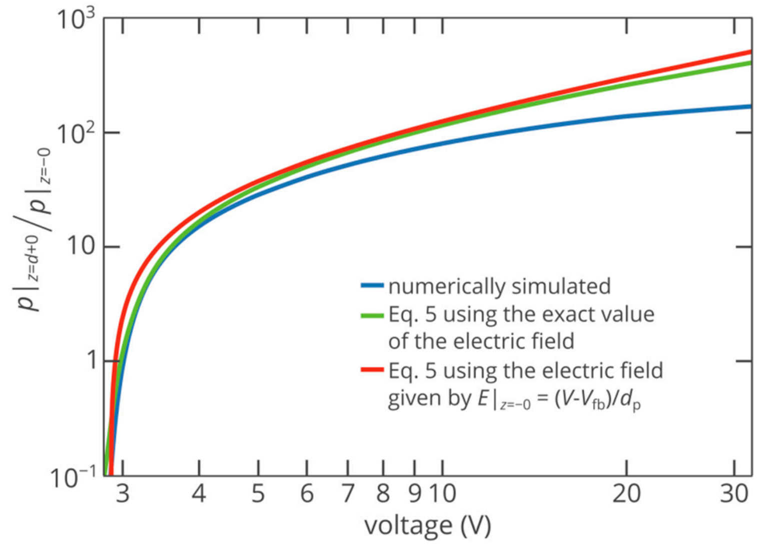

Figure S1. Dependence of the ratio of the hole density at the i-n junction to the hole density at the p-i junction on the bias voltage in the 4H-SiC p-i-n diode shown in Figure 2a in the main text. Here, we assume the recombination rate in the i-region of the p-i-n diode is equal to zero, while the recombination rate in the n-type and p-type layers is simulated using the parameters listed in Table S1. The blue curve is obtained from the self-consistent numerical simulations; the green curve is obtained using equation (5) and the electric field in the p-type region retrieved from the numerical simulations; the red curve is obtained using only equation (5) and the material parameters of 4H-SiC

listed in Table S1, the electric field in the p-type layer near the p-i junction is calculated using a very simple expression $E|_{z=0} = (V-V_{fb})/d_p$, where d_p is the size of the p-type region and $qV_{fb} = E_g + kT\times[\mathcal{F}_{1/2}^{-1}(n_{eqn}/N_c) + \mathcal{F}_{1/2}^{-1}(p_{eqp}/N_v)]$, where n_{eqn} and p_{eqp} are the electron and hole densities in the n-type and p-type injection layers, N_c and N_v are the effective densities of states in the conduction and valence bands of 4H-SiC, q is the electron charge, kT is the thermal energy, E_g is the bandgap energy of 4H-SiC and $\mathcal{F}_{1/2}^{-1}(x)$ is the inverse Fermi-Dirac function.

References

1. M. E. Levinshtein, S. L. Rumyantsev, and M. S. Shur, *Properties of Advanced Semiconductor Materials: GaN, AIN, InN, BN, SiC, SiGe* (John Wiley & Sons, 2001).
2. H. E. Ruda and B. Lai, "Electron transport in ZnS," J. Appl. Phys. **68**, 1714–1719 (1990).
3. I. Vurgaftman and J. R. Meyer, "Band parameters for nitrogen-containing semiconductors," J. Appl. Phys. **94**, 3675–3696 (2003).
4. L. Patrick and W. J. Choyke, "Static Dielectric Constant of SiC," Phys. Rev. B Condens. Matter **2**, 2255–2256 (1970).
5. C. Persson and U. Lindefelt, "Relativistic band structure calculation of cubic and hexagonal SiC polytypes," J. Appl. Phys. **82**, 5496–5508 (1997).
6. Collaboration: Authors and editors of the volumes III/17A-22A-41A1a, "Aluminum nitride (AlN) dielectric constants," in *Group IV Elements, IV-IV and III-V Compounds. Part a - Lattice Properties*, O. Madelung, U. Rössler, and M. Schulz, eds., Landolt-Börnstein - Group III Condensed Matter (Springer-Verlag, 2001), Vol. a, pp. 1–5.
7. N. T. Son, W. M. Chen, O. Kordina, A. O. Konstantinov, B. Monemar, E. Janzén, D. M. Hofman, D. Volm, M. Drechsler, and B. K. Meyer, "Electron effective masses in 4H SiC," Appl. Phys. Lett. **66**, 1074–1076 (1995).
8. R. Kaplan, R. J. Wagner, H. J. Kim, and R. F. Davis, "Electron cyclotron resonance in cubic SiC," Solid State Commun. **55**, 67–69 (1985).
9. N. T. Son, P. N. Hai, W. M. Chen, C. Hallin, B. Monemar, and E. Janzén, "Hole Effective Masses in 4H SiC Determined by Optically Detected Cyclotron Resonance," Mater. Sci. Forum **338-342**, 563–566 (2000).
10. W. J. Choyke, H. Matsunami, and G. Pensl, eds., *Silicon Carbide Recent Major Advances* (Springer, 2004).
11. O. Madelung, *Semiconductors: Other than Group IV Elements and III–V Compounds* (Springer Science & Business Media, 2012).
12. M. Suzuki and T. Uenoyama, "Strain effect on electronic and optical properties of GaN/AlGaN quantum-well lasers," J. Appl. Phys. **80**, 6868–6874 (1996).
13. Y. Taniyasu, M. Kasu, and T. Makimoto, "An aluminium nitride light-emitting diode with a wavelength of 210 nanometres," Nature **441**, 325–328 (2006).
14. M. Shur, *SiC Materials and Devices* (World Scientific, 2007).
15. D. Menichelli, M. Scaringella, F. Moscatelli, M. Bruzzi, and R. Nipoti, "Characterization of energy levels related to impurities in epitaxial 4H-SiC ion implanted p+n junctions," Diam. Relat. Mater. **16**, 6–11 (2007).
16. G. L. Harris, *Properties of Silicon Carbide* (IET, 1995).
17. R. H. Bube and Others, *Photoconductivity of Solids* (RE Krieger Pub. Co., 1978).
18. K. Takahashi, A. Yoshikawa, and A. Sandhu, *Wide Bandgap Semiconductors: Fundamental Properties and Modern Photonic and Electronic Devices* (Springer Science & Business Media, 2007).
19. A. O. Evwaraye, S. R. Smith, and W. C. Mitchel, "Shallow and deep levels in n-type 4H-SiC," J. Appl. Phys. **79**, 7726–7730 (1996).
20. S. Shionoya, W. M. Yen, and H. Yamamoto, *Phosphor Handbook* (Taylor & Francis, 2006).
21. Y. Taniyasu, M. Kasu, and T. Makimoto, "Electrical conduction properties of n-type Si-doped AlN with high electron mobility (>100cm2V−1s−1)," Appl. Phys. Lett. **85**, 4672–4674 (2004).
22. T. T. Mnatsakanov, M. E. Levinshtein, L. I. Pomortseva, and S. N. Yurkov, "Carrier mobility model for simulation of SiC-based electronic devices," Semicond. Sci. Technol. **17**, 974–977 (2002).
23. J. Pernot, W. Zawadzki, S. Contreras, J. L. Robert, E. Neyret, and L. Di Cioccio, "Electrical transport in n - type 4H silicon carbide," J. Appl. Phys. **90**, 1869–1878 (2001).
24. M. Roschke and F. Schwierz, "Electron mobility models for 4H, 6H, and 3C SiC [MESFETs]," IEEE Trans. Electron Devices **48**, 1442–1447 (2001).

25. N. Lophitis, A. Arvanitopoulos, S. Perkins, and M. Antoniou, "TCAD Device Modelling and Simulation of Wide Bandgap Power Semiconductors," in *Disruptive Wide Bandgap Semiconductors, Related Technologies, and Their Applications*, Y. K. Sharma, ed. (InTech, 2018).
26. O. Osamu, *Compound Semiconductor Bulk Materials And Characterizations* (World Scientific, 2012).
27. I. A. Khan and J. A. Cooper, "Measurement of high-field electron transport in silicon carbide," IEEE Trans. Electron Devices **47**, 269–273 (2000).
28. S. Adachi, *Properties of Semiconductor Alloys: Group-IV, III-V and II-VI Semiconductors* (John Wiley & Sons, 2009).
29. V. Grivickas, G. Manolis, K. Gulbinas, K. Jarašiūnas, and M. Kato, "Excess carrier recombination lifetime of bulk n-type 3C-SiC," Appl. Phys. Lett. **95**, 242110 (2009).
30. D. N. Bose, R. K. Ahrenkiel, and S. Bhunia, "Steady-state and time-resolved photoconductivity measurements of minority carrier lifetime in ZnTe," Journal of Applied Physics **86**, 6599–6601 (1999).
31. T. Koida, S. F. Chichibu, A. Uedono, A. Tsukazaki, M. Kawasaki, T. Sota, Y. Segawa, and H. Koinuma, "Correlation between the photoluminescence lifetime and defect density in bulk and epitaxial ZnO," Appl. Phys. Lett. **82**, 532–534 (2003).
32. M. Ohishi, "Time-Resolved Studies on Recombination Luminescence of Donor-Acceptor Pairs in ZnSe," Japanese Journal of Applied Physics **25**, 1546–1551 (1986).
33. B.-C. Lin, K.-J. Chen, C.-H. Wang, C.-H. Chiu, Y.-P. Lan, C.-C. Lin, P.-T. Lee, M.-H. Shih, Y.-K. Kuo, and H.-C. Kuo, "Hole injection and electron overflow improvement in InGaN/GaN light-emitting diodes by a tapered AlGaN electron blocking layer," Opt. Express **22**, 463–469 (2014).
34. Z. Hu, K. Nomoto, B. Song, M. Zhu, M. Qi, M. Pan, X. Gao, V. Protasenko, D. Jena, and H. G. Xing, "Near unity ideality factor and Shockley-Read-Hall lifetime in GaN-on-GaN p-n diodes with avalanche breakdown," Appl. Phys. Lett. **107**, 243501 (2015).
35. W. Wang, A. S. Lin, J. D. Phillips, and W. K. Metzger, "Generation and recombination rates at ZnTe:O intermediate band states," Appl. Phys. Lett. **95**, 261107 (2009).
36. A. V. Dmitriev and A. L. Oruzheinikov, "Radiative Recombination Rates in GaN, InN, AlN and their Solid Solutions," MRS Proc. **423**, 1832 (1996).
37. S. Selberherr, *Analysis and Simulation of Semiconductor Devices* (1984).
38. G. Liaugaudas, D. Dargis, P. Kwasnicki, R. Arvinte, M. Zielinski, and K. Jarašiūnas, "Determination of carrier lifetime and diffusion length in Al-doped 4H–SiC epilayers by time-resolved optical techniques," J. Phys. D Appl. Phys. **48**, 025103 (2015).
39. A. Udal and E. Velmre, "Investigation of Charge Carrier Lifetime Temperature-Dependence in 4H-SiC Diodes," Materials Science Forum **556-557**, 375–378 (2007).
40. A. Udal and E. Velmre, "Measurement of Charge Carrier Lifetime Temperature-Dependence in 4H-SiC Power Diodes," Materials Science Forum **338-342**, 781–784 (2000).
41. "3.3.3 High-Field Mobility and Velocity Saturation," http://www.iue.tuwien.ac.at/phd/ayalew/node67.html.
42. S. Potbhare, N. Goldsman, A. Lelis, J. M. McGarrity, F. Barry McLean, and D. Habersat, "A Physical Model of High Temperature 4H-SiC MOSFETs," IEEE Transactions on Electron Devices **55**, 2029–2040 (2008).

© 2019 by the authors. Submitted for possible open access publication under the terms and conditions of the Creative Commons Attribution (CC BY) license (http://creativecommons.org/licenses/by/4.0/).
